# Stigma, Post-traumatic Stress, and COVID-19 Vaccination Intent in Mongolia, India, and the United States

**DOI:** 10.3390/ijerph20032084

**Published:** 2023-01-23

**Authors:** David N. Sattler, Boldsuren Bishkhorloo, Kendall A. Lawley, Ruth Hackler, Chuluunbileg Byambajav, Michidmaa Munkhbat, Brooklyn Smith-Galeno

**Affiliations:** 1 Department of Psychology, Western Washington University, Bellingham, WA 98225-9172, USA; 2 Department of Education and Psychology, National University of Mongolia, Ulaanbaatar 14200, Mongolia; 3 Global Center for Integrated Health of Women, Adolescents, and Children, University of Washington, Seattle, WA 98195, USA

**Keywords:** COVID-19, stigma, post-traumatic stress, vaccination, Mongolia, India, United States

## Abstract

*Background*: Stigma and discrimination during the coronavirus (COVID-19) pandemic have increased precipitously worldwide. This multinational study examines how stigma, blaming groups for virus spread, concern regarding contracting the virus, resource loss, life satisfaction, and protective behaviors that help control the spread of COVID-19 are associated with post-traumatic stress and vaccine intent in Mongolia, India, and the United States. *Method*: 1429 people in Mongolia, India, and the United States completed measures assessing stigma during the COVID-19 pandemic, post-traumatic stress, blame, protective behaviors, and vaccine intent. *Results*: Mean post-traumatic stress scores in all three countries exceeded the cut-off that is commonly used to determine probable post-traumatic stress. Post-traumatic stress was associated with COVID-19 stigma experience, personal behavior change due to COVID-19 stigma, blaming groups for the spread of COVID-19, fear of COVID-19, and resource loss. In India and the United States, personal behavior change due to COVID-19 stigma, anger at individuals spreading COVID-19, and perceived susceptibility to illness were positively associated with vaccine intent. *Conclusions*: Stigma is a collateral stressor during the pandemic. The findings underscore the importance of prompt action to address stigma as a deleterious consequence of the pandemic. The findings illuminate potential barriers to receiving the vaccine and provide direction for future research to address barriers.

## 1. Introduction

Stigma and discrimination during the coronavirus (COVID-19) pandemic have increased precipitously worldwide. Healthcare professionals, persons who have tested positive for COVID-19, immigrants, and Asians have reported experiencing greater levels of stigma, physical and verbal abuse (e.g., physical altercations and attacks, discriminatory statements), home eviction, and alienation from communities due to the perception that they may be responsible for spreading the disease [1,2,3,4]. In addressing this pressing issue, United Nations Secretary-General António Guterres stated that: “COVID-19 does not care who we are, where we live, or what we believe. Yet the pandemic continues to unleash a tsunami of hate and xenophobia, scapegoating and scaremongering… I’m appealing for an all-out effort to end hate speech globally” [5]. Stigma, labeling, devaluing, and discrimination during the pandemic may be due, in part, to a fear that certain individuals pose a threat due to virus spread, lack of confidence in public health prevention measures to reduce or prevent virus transmission (e.g., vaccination or mask-wearing), and reports that the virus originated in China [6,7,8].

Stigma involves labeling, stereotyping, discrediting, and subjecting individuals to discrimination, based on a trait or characteristic that society perceives as a threat or has rejected [9,10]. Anticipated stigma involves the anticipation or fear of experiencing enacted stigma—the adverse attitudes and behaviors that are received from others—and can result from past experiences of being stigmatized by others or having an attribute or identity that is socially stigmatized [11,12,13]. Additionally, anticipated stigma may result when individuals identify with negative stereotypes and apply them to themselves [14].

Anticipated and enacted stigma are associated with psychological distress, low self-esteem, and feelings of shame in a variety of public health matters [10,15,16]. Prior to COVID-19, research examining stigma associated with severe acute respiratory syndrome (SARS) at the start of the 21st century identified several associated outcomes, such as anxiety and depression, independent of infection status [17]. HIV-related stigma was shown to contribute to psychological distress, even after accounting for health status and HIV-related symptoms [18,19]. During the COVID-19 pandemic, stigma has been associated with a variety of adverse outcomes, including stress, sleep disorders, anxiety, and depression [15,20]. Prolonged elevated levels of stress may further contribute to the undermining of psychological well-being [15,21,22,23,24]. Notably, individuals who experience COVID-related stigma may blame themselves for others becoming ill or dying, which can exacerbate feelings of anxiety and tension [25].

Stigma can serve as a barrier to engaging in health-related behaviors, including participating in testing for a virus and seeking treatment [26,27,28]. For example, in an attempt to avoid COVID-19 stigma or the perception that they are responsible for the virus’s spread, some individuals may decline to be screened for the virus to minimize the chance that others will view them as a threat; they may perceive that the adverse social consequences are more immediate and severe than the diagnosis and potential health risks due to the virus [12,27]. Research also demonstrates that people who endorse greater stereotypes about a particular disease may view themselves as being at lower risk for contracting that disease, suggesting that greater endorsement of COVID-19 stigma may be associated with a lower intent to be vaccinated [29].

This multinational study examines the prevalence of COVID-19 stigma and its relationship with post-traumatic stress in three countries: the United States, India, and Mongolia; and intent to receive the COVID-19 vaccination in the United States and India. Such multinational studies are vital for understanding commonalities and differences in experiences during the global pandemic, and for providing direction to develop interventions that address stigma and vaccine hesitancy. COVID-19-related stigma may be influenced by a host of factors, including the prevalence of the virus in a particular country, trust in and adherence to public health recommendations, healthcare capacity, availability of personal protective equipment (e.g., masks and disinfectant), quarantine policies, population size and density, economy, and culture [2,30,31].

In the United States, reports of stigma and violence directed at healthcare workers, Asian populations, and others increased during the pandemic [32]. The United States has had one of the highest COVID-19 case counts and death rates worldwide, and there has been significant debate concerning the utility and acceptance of public health measures to control virus spread (e.g., wearing masks, business closure, schools being taught online, vaccination requirements), trust in vaccine safety, and willingness to comply with public health mandates [33,34,35]. Some have argued that potential disparities in case counts and death rates may be due, in part, to differences in virus testing and reporting [36].

India, with a population nearly four times larger than the United States, also ranks among those countries reporting the highest number of COVID-19 cases and deaths [33,37]. Cultural values of interdependence among families and friends and close physical proximity in public spaces have made social distancing difficult [38]. As in the United States, COVID-19 illness and mortality have been attributed, in part, to comorbid conditions such as diabetes, cardiovascular disease, and hypertension [38]. Pre-existing tensions regarding race and class have been exacerbated during the pandemic, with widespread stigmatization of individuals who contracted the infection, including medical doctors and those from marginalized groups [39]. Further, greater economic hardship due to the pandemic in India was associated with individuals being less likely to acknowledge the civil and human rights of a victim of a COVID-19-related physical assault [1]. Research is needed to examine the extent of such reactions worldwide. There have been increases in reports of physical attacks during the pandemic, including on Muslims and healthcare workers [40].

Mongolia, with a population of 3.3 million, was one of the first countries to close its borders to prevent the spread of COVID-19 and to initiate a variety of public health measures, enhance risk communication, and establish a culture of community solidarity and engagement [41]. As a result of these and other measures, Mongolia reported only two COVID-19-related deaths and few if any instances of COVID-19-related stigma during the first year of the pandemic [37].

Building on existing stigma and mental health literature, we investigated how demographic variables, anticipated stigma, psychological distress, blaming groups for virus spread, concern regarding contracting the virus, resource loss, life satisfaction during the pandemic, and protective health behaviors that help control the spread of COVID-19 are associated with post-traumatic stress and vaccine intent [42,43]. Based on the research presented above, we expected that anticipated stigma would be positively associated with post-traumatic stress, but negatively associated with intent to vaccinate for COVID-19. We did not have specific predictions as to how these associations might vary across countries.

## 2. Method

### 2.1. Participants

The total number of participants was 1429 people (636 women, 725 men, and 5 unreported) in Mongolia, India, and the United States.

Mongolia. The participants were 629 persons (349 women, 280 men; age: *M* = 38, *SD* = 13, range: 18–80 years). Most had some college education or a college degree (70%), followed by secondary school completion (27%), and less than a secondary school education (2%). Most were married (61%), followed by single (34%), divorced/separated (4%), and other situation (1%).

India. The participants were 400 people (115 women, 218 men, 4 unreported; age: *M* = 31, *SD* = 7, range: 21–65 years). Most had some college education or a college degree (93%), followed by secondary school completion (2%), and less than a secondary school education (3%). Most were married (81%), followed by single (18%), and divorced/separated (1%).

United States. The participants were 400 people (172 women, 227 men, 1 unreported; age: *M* = 41, *SD* = 12, range: 18–72 years). Most had some college or a college degree (87%), followed by secondary school completion (12%), and less than a secondary school education (2%). Most were married (62%), followed by single (31%), and divorced/separated (7%).

### 2.2. Assessment Instruments

COVID-19 Stigma. We wrote 13 items to assess personal experience with COVID-19 stigma and behavior change due to perceived COVID-19 stigma. Examples of items include “People have avoided me because they think I have the coronavirus”, “Someone harassed me verbally because they think I have the coronavirus”, and “I wear a mask so people do not think I can spread the coronavirus”. Participants used a 5-point scale (1 = not at all to 5 = very much) to indicate their answers.

Post-traumatic Stress (PTS). The 20-item PTSD Checklist for DSM-5 (PCL-5) assessed symptoms associated with post-traumatic stress experienced in the past month [44]. Examples of items include “Repeated, disturbing, and unwanted memories of the coronavirus pandemic”, “Avoiding memories, thoughts, or feelings related to the coronavirus pandemic”, and “Irritable behavior, angry outbursts, or acting aggressively”. Participants used a 5-point scale (from 1 = not at all to 5 = extremely) to indicate their answers. High scores indicate greater stress. Reliability was excellent: Mongolia, α = 0.86; India, α = 0.96; United States, α = 0.98.

COVID-19 Fear and Illness Susceptibility. Six items asked about fear and anxiety about contracting the COVID-19 virus, based on the Fear of the COVID-19 scale [45]. Examples of items include “I am afraid of getting the coronavirus”, and “I am afraid of losing my life or becoming seriously ill because of the coronavirus”. Reliability was excellent: Mongolia, α = 0.82; India, α = 0.84; United States, α = 0.91. Two items asked about vulnerability to illness. An example is “Feeling that I am susceptible to infectious diseases”.

Minimizing the Threat of COVID-19. We wrote four items to assess minimizing the threat of COVID-19. Examples include “Keeping businesses open in my community is more important than preventing the spread of the coronavirus”, and “It’s more important for children to attend school in person than preventing the spread of the coronavirus”. Reliability was very good to excellent: Mongolia, α = 0.69; India, α = 0.83, and United States α = 0.92. Participants used a 5-point scale (1 = not at all to 5 = very much) to indicate their answers.

COVID-19 Self-Protection. We wrote four items to assess personal choice regarding actions to take to minimize the spread of COVID-19. Examples of items include “I should have the right to choose whether I want to comply with recommendations to prevent the spread of the coronavirus”, and “I should have the choice about what to do to protect myself from the coronavirus”. Participants used a 5-point scale (1 = not at all, to 5 = very much) to indicate their answers.

Resource Loss. Eighteen items assessed resource loss or the threat to resources due to COVID-19 [22,46]. Examples of items include sense of optimism, adequate income, companionship, time for adequate sleep, and motivation to get things done. Participants used a 5-point scale (0 = no loss to 4 = great loss) to indicate their answers. Higher scores indicate greater loss. Reliability was excellent: Mongolia, α = 0.89; India, α = 0.94; United States, α = 0.97.

Life Satisfaction. Seven items asked about life satisfaction during the pandemic. Examples are “Feeling satisfied with what I am achieving in life”, and “Feeling satisfied with my standard of living”. Participants used a 5-point scale (1 = not at all, to 5 = very much) to indicate their answers. Reliability was excellent: Mongolia, α = 0.83; India, α = 0.85; United States, α = 0.87.

Vaccine Intent. One item asked about the intent to receive a COVID-19 vaccine. Participants used a 5-point scale (1 = not at all, to 5 = very much) to indicate their answers. Participants in India and the United States completed this item (due to a procedural issue, it was not assessed in Mongolia).

### 2.3. Procedure

The study was approved by the Human Subjects Research Committee at Western Washington University and followed the American Psychological Association ethical guidelines. Participants in all countries completed the survey within the same one-week period, approximately one year after the pandemic was declared.

The participants in India and the United States were recruited using the crowdsourcing website Amazon Mechanical Turk (MTurk). Participants responded to a study titled “Coronavirus Responses” and received financial compensation for their participation. Conducting the study online during the pandemic was essential because it eliminated the possibility of virus spread between participant and researcher, and because of physical distancing measures that limited in-person interaction. Participants were required to report a MTurk reputation score of 0.90 or greater in order to ensure the quality of the data [47]. Numerous attention checks assessed whether participants were reading the items (e.g., “If you are reading this item, respond with strongly agree”). The survey took about 25 min to complete.

In Mongolia, because online crowdsourcing platforms such as MTurk are not available, we used the snowball nonprobability sampling technique and contacted individuals via the telephone. This approach was essential during the pandemic because it allowed us to conduct the study with no potential for virus spread between the participants and research assistants. The response rate was 98%, with nearly everyone agreeing to participate. The second author trained eight advanced National University of Mongolia psychology and sociology students (the research assistants) in questionnaire administration and research ethics. To translate the questionnaire into Mongolian, we used a version of the committee approach that we have successfully used in prior research examining post-traumatic stress responses in Mongolia and other countries [22,43,48,49,50]. Specifically, a professional translator translated the questionnaire, and the second, fifth, and sixth authors then met with the bilingual students online and reviewed the translation. They discussed any issues and made edits as appropriate. The research assistants read the questions to the participants, who reported the items were clear and understandable. It took about 30 min to complete the survey.

### 2.4. Data Analytic Plan

We first performed principal component factor analyses with varimax rotation and then assessed the reliability of the factors for each of the three samples. Next, for each sample we performed hierarchical regression analyses to examine the factors that predict post-traumatic stress and the intent to receive a COVID-19 vaccine. Based on prior research, we entered individual characteristics (gender, age, and education), followed by stigma and COVID-19 experiences (e.g., resource loss) [22,43]. Descriptive statistics are presented in Table 1.

## 3. Results

### 3.1. Factor Analyses

COVID-19 Stigma. Principal component factor analysis with varimax rotation was performed on the 13 stigma items. Three factors with eigenvalues greater than one and factor loadings greater than 0.57 emerged. Factor 1 assessed personal experience with COVID-19 stigma (6 items, Mongolia α = 0.78; India α = 0.90; United States α = 0.96). Factor 2 assessed personal behavior change due to COVID-19 stigma (3 items, Mongolia, α = 0.78; India, α = 0.60; United States, α = 0.66). Factor 3 assessed personal experience as being excluded due to COVID-19 stigma (2 items, Mongolia α = 0.60; India α = 0.83; United States α = 0.91).

COVID-19 Spread Blame. Principal component factor analysis with varimax rotation was performed on the nine items assessing COVID-19-related perception of others. Two factors with eigenvalues greater than one and factor loadings greater than 0.57 emerged. Factor 1 assessed blaming groups for spreading COVID-19 (2 items, Mongolia, α = 0.87; India, α = 0.79; United States, α = 0.85). Factor 2 assessed blaming people in general for spreading COVID-19 (3 items, Mongolia, α = 0.43; India, α = 0.56; United States, α = 0.71).

COVID-19 Self-Protection. Principal component factor analysis with varimax rotation was performed on the four COVID-19 self-protection items. Two factors with eigenvalues greater than one and factor loadings greater than 0.60 emerged. Factor 1 assessed the right to choose whether to protect oneself against COVID-19 (Mongolia, α = 0.82; India, α = 0.64; United States, α = 0.87). Factor 2 assessed negative behaviors in response to being directed to wear a mask to prevent COVID-19 spread (Mongolia, α = 0.62; India, α = 0.51; United States, α = 0.76).

### 3.2. Predicting Post-traumatic Stress

Table 1 presents the means and standard deviations for the PTSD Checklist for DSM-5 (PCL-5). A cut-off score between 31–33 is commonly used as a guide to evaluating probable post-traumatic stress. The mean for all countries exceeded the cut-off score, with the mean in India the highest, followed by the United States and Mongolia.

Mongolia. Table 1 shows the predictor variables explained 46% of the variance in post-traumatic stress during the COVID-19 pandemic, *F*(21, 584) = 25.13, *p* < 0.0001. Post-traumatic stress was positively associated with being female, personal experience with COVID-19 stigma, personal behavior change due to COVID-19 stigma, blaming other groups for COVID-19 spread, anger at individuals spreading COVID-19, belief one is susceptible to illness, COVID-19 fear, lack of agency, minimizing COVID-19 threat, performing behaviors to minimize the spread of COVID-19, and resource loss. Post-traumatic stress was negatively associated with life satisfaction.

India. Table 1 shows the predictor variables explained 82% of the variance in post-traumatic stress during the COVID-19 pandemic, *F*(21, 354) = 83.27, *p* < 0.0001. Post-traumatic stress was positively associated with personal experience with COVID-19 stigma, personal behavior change due to COVID-19 stigma, stigma being excluded due to COVID-19 concern, blaming groups for the spread of COVID-19, anger at individuals spreading COVID-19, the belief that one is susceptible to illness, COVID-19 fear, lack of agency, and resource loss. Post-traumatic stress was negatively associated with the age and education level of participants.

United States. Table 1 shows the predictor variables explained 90% of the variance in post-traumatic stress during the COVID-19 pandemic, *F*(21, 375) = 165.02, *p* < 0.0001. Post-traumatic stress was positively associated with personal experience with COVID-19 stigma, personal behavior change due to COVID-19 stigma, stigma being excluded due to COVID-19 concerns, blaming other groups for the spread of COVID-19, COVID-19 fear, lack of agency, supporting governmental response, and resource loss. Post-traumatic stress was negatively associated with life satisfaction.

### 3.3. Predicting Vaccine Intent

India. Table 2 shows the predictor variables explained 21% of the variance in intention to receive the COVID-19 vaccine, *F*(17, 361) = 6.92, *p* < 0.0001. Intent to receive the COVID-19 vaccine was positively associated with personal experience with COVID-19 stigma, personal behavior change due to COVID-19 stigma, blaming other groups for the spread of COVID-19, anger at individuals spreading COVID-19, belief that one is susceptible to illness, COVID-19 fears, and COVID-19 self-protection. Intent to receive the vaccine was negatively associated with minimizing the threat of COVID-19.

United States. Table 2 shows the predictor variables explained 25% of the variance in intention to receive the COVID-19 vaccine, *F*(17, 379) = 9.12, *p* < 0.0001. Intent to receive the COVID-19 vaccine was positively associated with personal behavior change due to COVID-19 stigma, anger at individuals spreading COVID-19, belief one is susceptible to illness, and life satisfaction. Intent to receive the vaccine was negatively associated with lack of agency and COVID-19 self-protection.

## 4. Discussion

This multinational study investigated the relationships among COVID-19 stigma and post-traumatic stress in Mongolia, India, and the United States, and with vaccination intention in India and the United States. The findings indicate significant mental health consequences related to COVID-19 stigma. Mean post-traumatic stress scores in all three countries exceeded the cut-off that is commonly used to determine probable post-traumatic stress, with India scoring the highest mean, followed by the United States and Mongolia. Post-traumatic stress was associated with personal experience with COVID-19 stigma, personal behavior change due to COVID-19 stigma, blaming groups for the spread of COVID-19, fear of COVID-19, lack of agency, and resource loss in each location.

We first discuss the findings in each country, and then consider the implications of the findings. In India, personal experiences with exclusion due to COVID-19 stigma, anger at individuals spreading COVID-19, and susceptibility to illness predicted post-traumatic stress. Reports of COVID-19 stigmatization in India indicate that healthcare workers were forced out of their homes and communities, abandoned by family members, and attacked for providing medical care [51]. Similarly, anger toward others believed to be spreading COVID-19 predicted post-traumatic stress. Notably, research during the pandemic reports that fear and anxiety concerning the pandemic may trigger feelings of anger [52]. Blaming others may serve to promote feelings of control when confronted with a threatening situation [52,53]. Further, anecdotal evidence suggests that such anger may stem from feeling that other people are responsible for prolonging the pandemic [52]. Perceptions of susceptibility to illness may be related to post-traumatic stress, due to the high rates of disease from other viruses that can be fatal when they interact with COVID-19. COVID-19 morbidity and mortality in India have been attributed, in part, to co-morbid conditions, including diabetes, hypertension, and cardiovascular disease; individuals with these conditions may be especially concerned about contracting COVID-19 [38].

In Mongolia, susceptibility to illness, anger at individuals spreading COVID-19, minimizing the COVID-19 threat, and life satisfaction were associated with post-traumatic stress. Feelings of anger may stem, in part, from concern that others are not taking appropriate measures to stop the spread of COVID-19 [52]. Those feeling susceptible to illness may be more likely to express higher levels of post-traumatic stress [54,55]. Interestingly, the findings show that minimizing the COVID-19 threat was positively associated with post-traumatic stress in Mongolia. This finding is contrary to prior work, that found the perceived threat of COVID-19 predicted increased post-traumatic stress [56]. One explanation may be that other factors not measured in this study, such as the degree of social support and quality of life, may mediate the effect of minimizing the COVID-19 threat and post-traumatic stress. For example, low levels of support from family and friends may be associated with psychological distress, while negating any possible protective benefits of minimizing the threat of COVID-19 [57]. However, additional research is needed to explore this finding. Finally, life satisfaction in Mongolia was negatively associated with experiencing post-traumatic stress. These findings are consistent with previous literature, which demonstrated that greater access to resources, including emotional and psychological resources, can act as a buffer against post-traumatic stress when systemic stressors are present [22].

In the United States, personal experience with exclusion due to COVID-19 stigma and life satisfaction were associated with post-traumatic stress. As with the findings in India, experiencing exclusion due to COVID-19 stigma and pervasive stress may lead to feelings of social worthlessness [58]. Additionally, physical distancing efforts have been implemented in many parts of the country, which prevented people from seeing loved ones for months at a time. At a time when people were already feeling distanced from those that they cared about, experiencing social exclusion or social rejection due to COVID-19 stigma may be especially difficult. Similar to the findings in Mongolia, life satisfaction was negatively associated with post-traumatic stress. These findings are consistent with research examining the relationship between the availability of resources and post-traumatic stress, such that greater access to resources during the pandemic may act as a protective factor against post-traumatic stress in a variety of cultural contexts [59,60].

Taken together, the findings of this multinational study show that stigma is a significant collateral stressor during the pandemic. In the midst of a global disaster, where individuals have felt isolated and alone, being stigmatized and ostracized by others can be especially harmful to psychological well-being. Because COVID-19 has been a constant threat to health and safety, people may also stigmatize others in an attempt to protect themselves and their loved ones from this persistent and life-threatening disease. With this in mind, the widespread availability of the COVID-19 vaccine, in addition to other self-protective behaviors, such as mask-wearing, physical distancing, and handwashing, may help in reducing perceptions of the COVID-19 threat and stigma. In addition, the findings underscore the mental health consequences of resource loss experienced during the pandemic [61]. Research examining ways to reduce loss and promote resource gains and mental health during the pandemic, especially in consideration of elevated post-traumatic stress scores shown in this study, is warranted.

Regarding vaccine intent, the findings in both India and the United States show that personal behavior change due to COVID-19 stigma, anger at individuals spreading COVID-19, and perceived susceptibility to illness were positively associated with vaccine intent. One possible explanation for the relationship between behavior change due to stigma and vaccine intent may be that receiving the vaccine, in addition to providing protection from the virus, might serve as a mechanism to reduce the likelihood of others perceiving the individual as a threat, and thus reduce perceived stigmatized status. In other cases, individuals strictly adhering to COVID-19 safety recommendations may feel anger toward others perceived to be spreading COVID-19 by not following safety precautions. The COVID-19 vaccine provided many with the opportunity to re-engage in activities that were commonplace before the pandemic, and thus may reduce feelings of anger toward others [62]. In short, these findings may help identify potential barriers to receiving the vaccine and thus provide direction to breaking down barriers.

There are a few limitations to the study. We were not able to employ a pure random sampling strategy. MTurk was available in India and the United States, but MTurk or a similar online platform was not available in Mongolia. While comparisons across countries are instructive, responses may have been influenced by within-country events or other variables that were not assessed. We do not know how these or other variables, such as social desirability, may have influenced responses. The high quality of the sample is reflected in both the high response rate in Mongolia (98%) and our requirement that participants in India and the United States have a high MTurk reputation score, which has been shown to produce high-quality samples [47]. The findings may not generalize to the populations of each country. We were not able to assess pre-pandemic psychopathology or other experiences, and we do not know how experiences within each country during the pandemic may have influenced responses to and scores on the post-traumatic stress measure. Research is needed to examine in more detail the contributions of other experiences during the pandemic to stress response. Other measures of stress response beyond those assessed in this study may provide valuable information about experiences during the pandemic. The study relied on self-report and was correlational, but self-report data during large-scale community-wide stressors appears to be reliable [63]. As the pandemic and vaccine options evolve, research is needed to examine barriers to receiving the vaccine, including stigma [64,65]. Finding ways to provide effective education about the importance of vaccination, especially for those who are uninterested, is essential in controlling the pandemic.

## 5. Conclusions

The findings provide valuable direction for mental health interventions, overcoming barriers, and promoting COVID-19 vaccination globally. Notably, the findings underscore the importance of taking prompt action to address the development of stigma as a deleterious consequence of the pandemic, as illustrated by the vital words of António Guterres, United Nations Secretary-General, as discussed above. It is likely that the development and distribution of COVID-19 vaccines had positive consequences for reducing stigma, and future research addressing this essential issue is warranted. Importantly, the findings help illuminate potential barriers to receiving the vaccine and provide direction for future research to examine ways to effectively address barriers.

Research examining factors that contribute to and reduce COVID-19 stigma is warranted [1]. The alarming worldwide increase in attacks on Asians during the pandemic is associated with reports that the virus originated in China and certain government leaders referring to it as the “China virus.” Research is needed to examine approaches to effectively mitigate such responses. Research is also needed to examine the role of cultural norms, family structure, and national approaches to managing and mitigating COVID-19 spread, and vaccination efforts, and their association with mental and physical health, as well as unique cultural expressions of distress [66].

## Figures and Tables

**Table 1:** Predicting post-traumatic stress during the COVID-19 pandemic in Mongolia, India, and the United States.

	Mongolia (*n* = 629)						India (*n* = 400)						United States (*n* = 400)					
Variable	*B*	*SE B*	β	Adj. *R*^2^	*M*	*SD*	*B*	*SE B*	β	Adj. *R*^2^	*M*	*SD*	*B*	*SE B*	β	Adj. *R*^2^	*M*	*SD*
Post-traumatic Stress					35.69	11.11					69.92	17.25					51.13	23.65
Post-traumatic Growth					2.92	0.99					4.07	0.87					3.15	1.36
Step 1: Demographics				0.02 ^c^						0.25 ^c^						0.00		
Gender	3.09	0.90	0.14 ^c^		-	-	1.32	1.74	0.03		-	-	0.69	2.48	0.01		-	-
Age	−0.06	0.04	−0.07		38	13	−1.21	0.12	−0.47 ^c^		31	7	−0.17	0.11	−0.08		41	12
Education	−0.051	0.32	−0.07		-	-	−4.14	1.02	−0.18 ^c^		-	-	2.01	1.50	0.07		-	-
Step 2: Stigma				0.04 ^c^						0.71 ^c^						0.78 ^c^		
Experience	3.16	1.30	0.10 ^a^		1.12	0.36	7.84	0.87	0.51 ^c^		3.17	1.12	6.76	1.12	0.39 ^c^		2.17	1.36
Behavior Change	1.18	0.35	0.14 ^c^		3.17	1.26	4.88	0.71	0.23 ^c^		3.52	0.82	2.37	0.65	0.11 ^c^		2.96	1.11
Exclusion	2.76	1.73	0.07		1.05	0.26	2.05	0.79	0.15 ^b^		3.16	1.23	7.25	0.99	0.44 ^c^		2.14	1.43
Step 3: Blame for Spread				0.10 ^c^						0.74						0.78		
Blaming groups	2.47	1.06	0.09 ^c^		1.13	0.42	3.59	0.83	0.20 ^c^		3.24	0.96	1.49	0.75	0.07 ^a^		2.57	1.18
General anger	2.39	0.55	0.18 ^c^		2.57	0.86	2.65	0.70	0.12 ^c^		3.62	0.79	0.22	0.65	0.01		3.20	1.11
Step 4: Illness Concerns				0.23 ^c^						0.79 ^c^						0.82 ^c^		
Susceptibility to Illness	1.89	0.37	0.19 ^c^		1.87	0.98	1.63	0.50	0.09 ^c^		3.36	0.90	−0.04	0.57	0.00		3.08	1.15
COVID-19 Fear	3.78	0.51	0.31 ^c^		2.57	0.90	6.46	0.82	0.32 ^c^		3.46	0.86	7.03	0.80	0.33 ^c^		2.89	1.12
Step 5: Threat and Self-Protection				0.29 ^c^						0.80 ^c^						0.83 ^c^		
Others Control Decisions	2.52	0.39	0.24 ^c^				2.39	0.62	0.12 ^c^		3.11	0.87	1.75	0.54	0.09 ^c^		2.69	1.18
Minimizing the Threat	0.88	0.45	0.07 ^a^		2.53	0.93	0.00	0.79	0.00		3.32	0.99	0.64	0.82	0.04		2.55	1.32
COVID-19 Self-Protection	−0.09	0.29	−0.01		2.59	1.38	1.64	0.62	0.09 ^b^		3.45	0.92	0.25	0.67	0.01		2.69	1.34
Step 6: Life Satisfaction				0.34 ^c^						0.80 ^b^	3.29	0.68				0.83 ^b^	3.28	0.88
Step 7: Resource Loss				0.44 ^c^	36.24	13.38				0.82 ^c^	59.56	14.15				0.90 ^c^	44.00	19.73

Notes: ^a^ = *p* < 0.05. ^b^ = *p* < 0.01. ^c^ = *p* < 0.001. B = unstandardized beta. SE B = standard error for the unstandardized beta. Β = standardized beta. Adj. *R*^2^ = adjusted R-squared.

**Table 2:** Predicting vaccine intent in India and the United States.

	India				United States			
Variable	*B*	*SE B*	β	Adj. *R*^2^	*B*	*SE B*	β	Adj. *R*^2^
Step 1: Demographics				0.00				0.01
Gender	−0.09	0.12	−0.04		−0.27	0.14	−0.10	
Age	0.00	0.01	−0.03		0.01	0.01	0.07	
Education	0.00	0.07	0.00		0.16	0.08	0.09	
Step 2: Stigma				0.08 ^c^				0.11 ^c^
Experience	0.27	0.09	0.31 ^b^		−0.09	0.12	−0.09	
Behavior Change	0.24	0.07	0.19 ^b^		0.48	0.07	0.41 ^c^	
Exclusion	−0.12	0.08	−0.15		−0.11	0.11	0.11	
Step 3: Blame for Spread				0.14 ^c^				0.19 ^c^
Blaming groups	0.19	0.09	0.18 ^a^		−0.11	0.08	0.10	
General anger	0.33	0.07	0.26 ^c^		0.42	0.07	0.35 ^c^	
Step 4: Illness Concerns				0.17 ^b^				0.19
Susceptibility to Illness	0.16	0.06	0.14 ^b^		0.15	0.07	0.13 ^a^	
COVID-19 Fear	0.19	0.04	0.16 ^a^		0.10	0.09	0.09	
Step 5: Threat and Self-Protection				0.19 ^b^				0.23 ^c^
Others Control Decisions	−0.10	0.07	−0.09		−0.14	0.06	−0.12 ^a^	
Minimizing the Threat	−0.20	0.09	−0.20 ^a^		−0.06	0.09	−0.06	
Self-Protection	0.17	0.07	0.15 ^a^		−0.19	0.08	−0.20 ^a^	
Step 6: Life Satisfaction				0.19				0.25 ^c^
Step 7: Resource Loss				0.19				0.25

Note: ^a^ = *p* < 0.05. ^b^ = *p* < 0.01. ^c^ = *p* < 0.001. B = unstandardized beta. SE B = standard error for the unstandardized beta. Β = standardized beta. Adj. *R*^2^ = adjusted R-squared.

## Data Availability

The data presented in this study are available on request from the corresponding author.
